# The underlying molecular mechanism and drugs for treatment in adrenal cortical carcinoma

**DOI:** 10.7150/ijms.60261

**Published:** 2021-06-16

**Authors:** Chengquan Ma, Jian Xiong, Hao Su, Hongjun Li

**Affiliations:** Department of Urology, Peking Union Medical College Hospital, Peking Union Medical College, Chinese Academy of Medical Sciences, Beijing, China.

**Keywords:** adrenal cortical carcinoma, biomarkers, prognosis, treatment

## Abstract

**Purpose**: The study aimed to predict and explore the possible clinical value and mechanism of genetic markers in adrenal cortical carcinoma using a bioinformatics analysis method.

**Methods**: The RNA-seqs and miRNAs data were downloaded from TCGA database to identify the differentially expressed genes and differentially expressed miRNAs. The hub-genes were screened by building protein-protein interaction sub-networks with 12 topological analysis methods. We conducted the receiver operating characteristic curve to elevate the diagnostic value of hub-genes in distinguishing the death and alive groups. The survival analysis of hub-genes and key miRNAs were conducted using Kaplan-Meier curves. Furthermore, most significant small molecules were identified as therapeutic candidates for adrenal cortical carcinoma by the CMap analysis.

**Results**: Compared to survival group, we found 475 up-regulated genes and 354 genes and the key pathways leading to the death of different ACC individual patients. Then we used 12 topological analysis methods to found the most possible 22 hub-genes. Among these hub-genes, nine hub-genes (C3, CXCL5, CX3CR1, GRM8, HCAR2, HTR1B, SUCNR1, PTGER3 and SSTR1) could be used to distinguish the death and survival groups for patients. We also revealed that mRNA expressions of 12 genes (C3, CXCL8, CX3CR1, GNAT3, GNGT1, GRM8, HCAR2, HTR1B, HTR1D, PTGER3, SSTR1 and SUCNR1) and four key miRNAs (hsa-mir-330, hsa-mir-489, hsa-mir-508 and hsa-mir-513b) were related to survival. Three most small molecules were identified (H-9, AZ-628 and phensuximide) as potential therapeutic drugs for adrenal cortical carcinoma.

**Conclusion**: The hub-genes expression was significant useful in adrenal cortical carcinoma, provide new diagnostic, prognosis and therapeutic approaches for adrenal cortical carcinoma. Furthermore, we also explore the possible miRNAs involved in regulation of hub-genes.

## Introduction

Adrenal cortical carcinoma (ACC) is a rare malignant tumor that evolves from the adrenal cortex, with an incidence worldwide of 0.7-2.0 cases/million/year 1. Despite a lot of clinical study, the prognosis is still very poor, with a 5-year survival rate of < 40% 2, 3. Prognostic factors are not known: there are at least 4 clinicopathological factors: ENSAT stage, resection (R0 vs R1/R2) status, Ki67 proliferation index, hormone hypersecretion 4. However, due to the small number of ACC cases available for study, the clinicopathological characteristics and prognostic factors of ACC are not very clear yet. Correspondingly, it is true that the only chance of cure is surgery and there is no effective treatment method for most patients to prolong survival 4. Therefore, it is necessary to look for the occurrence and development mechanism of ACC and therapeutic targets are particularly important.

Compared with other tumors, ACC is highly malignant and is characterized by a high mortality rate within 1-3 years of diagnosis. Around 30-40% of ACC have clear evidence of metastasis in clinical presentation, and the available systematic treatments rarely yield a complete remission 5, 6. Previous studies have focused on the studies of comparing normal and tumor, and related results suggest that its occurrence and development may be related to the overexpression of IGF-2, TP53 gene mutation and abnormal activation of the Wnt/13-catenin signaling pathway, but it is not related to the lethal aspects of this disease. ACC-specific literature is plenty of demonstrations that the above cited genetic alterations portend a poorer survival. Furthermore, more recent comprehensive genomic analyses of ACC 7-11 have gained significant improvements in our knowledge of this disease. In particular, the ACC-TCGA sub-project which represents the base of the present works 12. 3 patterns of gene regulation systems are described in cancer (copy number changes, differential microRNA expression and gene CpG methylation status) and we know that methylation has a role in ACC based on the findings of the above research. However, the specific lethal molecular mechanism of this disease is not clear though previous study also conducted many explorations in RNA expression and microRNA expression 13-15. Therefore, we downloaded RNA-seqs and miRNAs data from TCGA database using a bioinformatics analysis method to explore the possible clinical value and mechanism of genetic markers for ACC: a). identify the differentially expressed genes (DEGs) and differentially expressed miRNAs; b). building protein-protein interaction (PPI) sub-networks; c). conducted the receiver operating characteristic (ROC) curve to elevate the diagnostic value of hub-genes in distinguishing the death and alive groups; d). conducted survival analysis of hub-genes and key miRNAs; e). identified small molecules were as therapeutic candidates for ACC by the CMap analysis.

## Methods

### Data resources and sample grouping

Series matrix files associations with ACC were downloaded from TCGA. The normalized gene expression data format was FPKM. A total of 78 adults (>18 years) ACC were included in our study, which is divided into 51 alive samples and 27 dead samples according the results of follow up.

### Screening for DEGs and different miRNA

The matrix data of mRNA were performed log2 conversion and normalization using limma package of R/ Bioconductor software. The limma package was also utilized to screen and identify the DEGs between dead samples and alive samples. Adjust P values <0.01 and |log2FC| >2 were considered the statistical significance of differential expression. The matrix data of miRNA conducted method to reveal the different miRNAs between dead samples and alive sample, with adjust P value <0.05 and |log2FC| >1 was considered the statistical significance of differential miRNAs. The heat map and volcano map were also plotted for samples and identified DEGs / differential miRNAs with pheatmap package in R software.

### GO and KEGG enriched pathway analysis

To explore the underlying pathways and biological processes, we conducted GO and KEGG pathway analyses based on all differentially expressed mRNAs using the Database for Annotation Visualization and Integrated Discovery (DAVID version 6.8; https://david.ncifcrf.gov/).

### Establishment of PPI network and identification of hub-genes

A PPI network was established by the STRING (v11.0; https://string-db.org/) 16 and visualized by the Cytoscape 3.6.1. The hub-genes were screened by building PPI sub-networks with 12 topological analysis methods. Then we selected the top 15 genes for every topological analysis methods into the Venn plot method. The “Molecular Complex Detection” (MCODE), a clustering algorithm identifying locally densely connected regions in a large PPI network based on a node-weighting arithmetic, was employed to recognize highly interacted hub-genes clustering.

### Prognostic association between OS and the expression of hub-genes

We conducted the receiver operating characteristic (ROC) curve to elevate the diagnostic value of hub-genes in distinguishing the death and survival groups.

### Survival analysis of hub-gene

The Gene Expression Profiling Interactive Analysis (GEPIA) database was utilized to assess the impact of hub-genes on the patients' prognosis including overall survival (OS) and disease-free survival (DFS).

### Target mRNA prediction miRNA

We used the ENCORI database to predict the possible miRNAs that may regulate the hub-genes. Then choose the result of possible miRNAs was consistent with the differential miRNAs previously detected in the TCGA database. Finally, the most potentially miRNAs participate in the regulation of mRNAs were defined by the correlation analysis between them.

### Identification of small molecules

The CMap database (http://www.broadinstitute.org/cmap/) was used to explore potential small molecule drugs for use in patients based on the genes signature of ACC. The overlapping differently expressed genes based on top 5 modules were classified into up-regulated and down-regulated groups. The negative connectivity score (closer to -90) demonstrate greater similarity between the genes.

### Survival analysis of miRNA

The UALCAN (http://ualcan.path.uab.edu/analysis.html) database 17 was utilized to assess the impact of key miRNAs that regulates the hub-genes on the patients' prognosis.

## Results

### Patient characteristics

The study included 51 alive samples and 27 dead samples at a mean age of 45.96±15.21 years and 49.30±15.89 years, respectively (p=0.367). There is no difference between the two groups of gender (p=0.851), lymph node (p=0.69) and metastasis (p=0.09). It is significant difference between the two groups on stage (p=0.033).The ratio of patients with T3/T4 stage was more than T1/T2 stage in dead group (Table 1).

### Identification of DEGs

**Figure 1:**
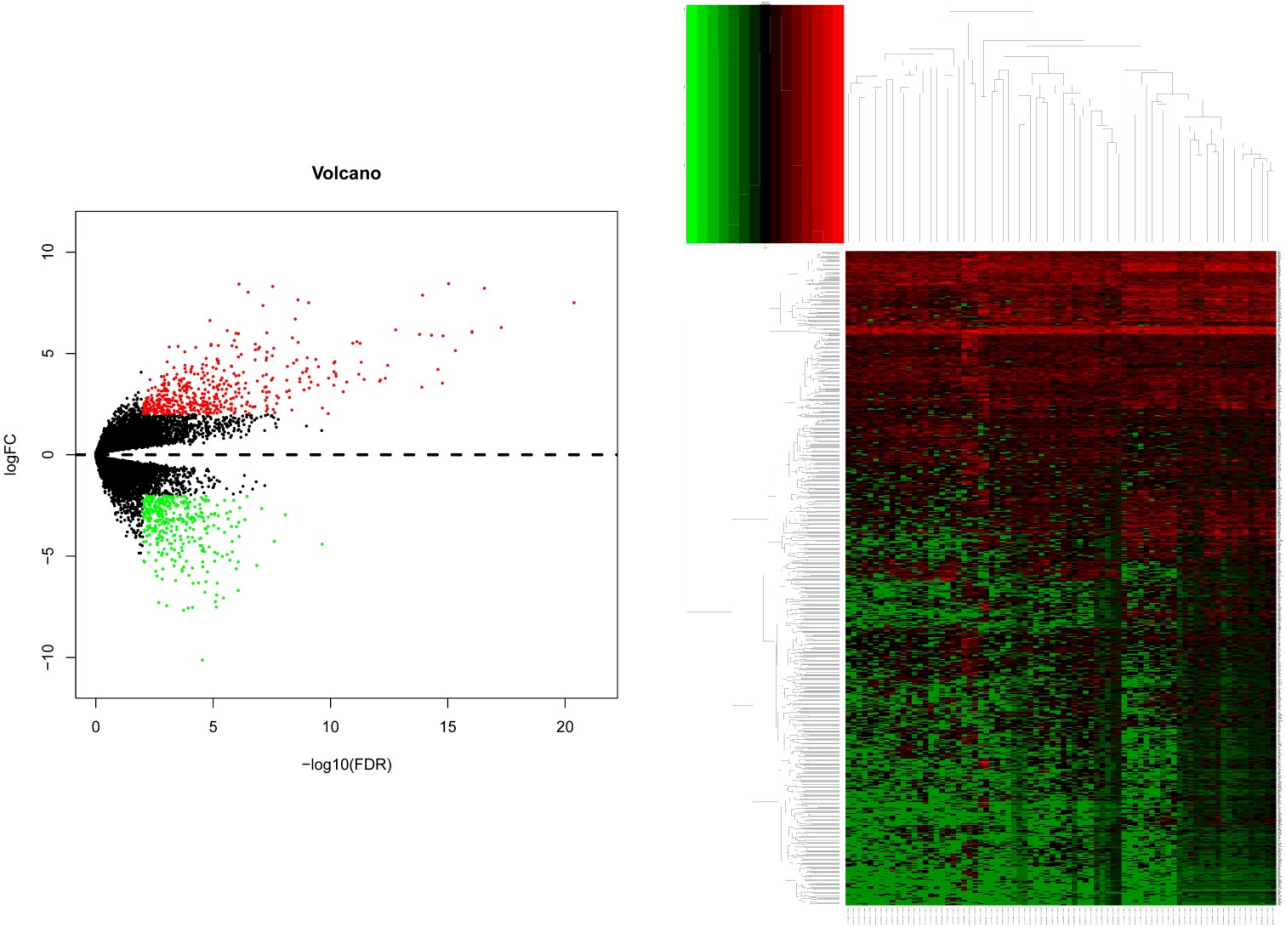
The volcano plot and heatmap of the distribution of DEGs. Comparison of gene expression profiles with dead samples and alive samples. Adjust P values <0.01 and |log2FC| >2 were considered the statistical significance of differential expression. 475 genes were up-regulated and 354 genes were down-regulated in dead group compared to alive group.

After gene differential expression analysis of microarray data, 475 genes were up-regulated and 354 genes were down-regulated in dead group compared to alive group. The volcano plot and heatmap of the distribution of DEGs is shown in Figure 1.

### Functional and pathway enrichment analyses

**Figure 2:**
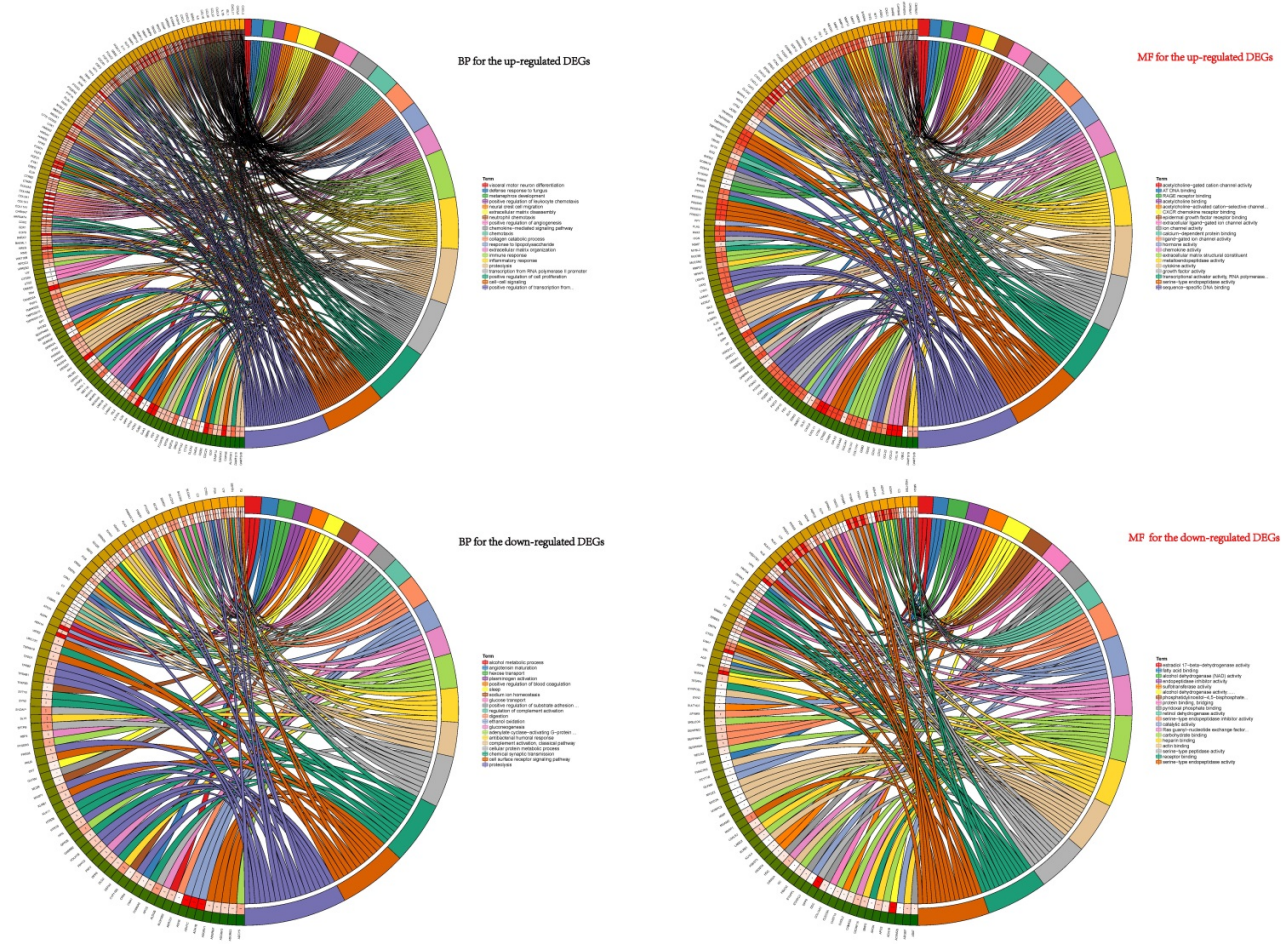
BP and MF analyses of the DEGs. BP showed that the up-regulated DEGs were mainly enriched in progress: cell-cell signaling, collagen catabolic process, chemokine-mediated signaling pathway, positive regulation of cell proliferation, inflammatory response and neutrophil chemotaxis et.al. These down-regulated genes participated in vital biological processes: ethanol oxidation, cellular protein metabolic process, antibacterial humoral response, hexose transport, adenylate cyclase-activating G-protein coupled receptor signaling pathway, alcohol metabolic process, proteolysis, gluconeogenesis, cell surface receptor signaling pathway, and chemical synaptic transmission plasminogen activation et.al. MF for the up-regulated and down-regulated DEGs were also conducted shown in right half of figure 2.

To explore the potential biological functions of the DEGs, GO terms (including BP and MF analyses) and KEGG pathway analysis were performed. GO pathways for BP showed that the up-regulated DEGs were mainly enriched in progress including cell-cell signaling, collagen catabolic process, chemokine-mediated signaling pathway, positive regulation of cell proliferation, inflammatory response, neutrophil chemotaxis, chemotaxis and immune response et al. (Figure 2). The GO analysis results revealed that these down-regulated genes participated in vital biological processes including ethanol oxidation, cellular protein metabolic process, antibacterial humoral response, hexose transport, adenylate cyclase-activating G-protein coupled receptor signaling pathway, alcohol metabolic process, proteolysis, gluconeogenesis, cell surface receptor signaling pathway, and chemical synaptic transmission plasminogen activation et al. (Figure 2). Functional analyses of MF for the up-regulated and down-regulated DEGs were also conducted (Figure 2).

**Figure 3:**
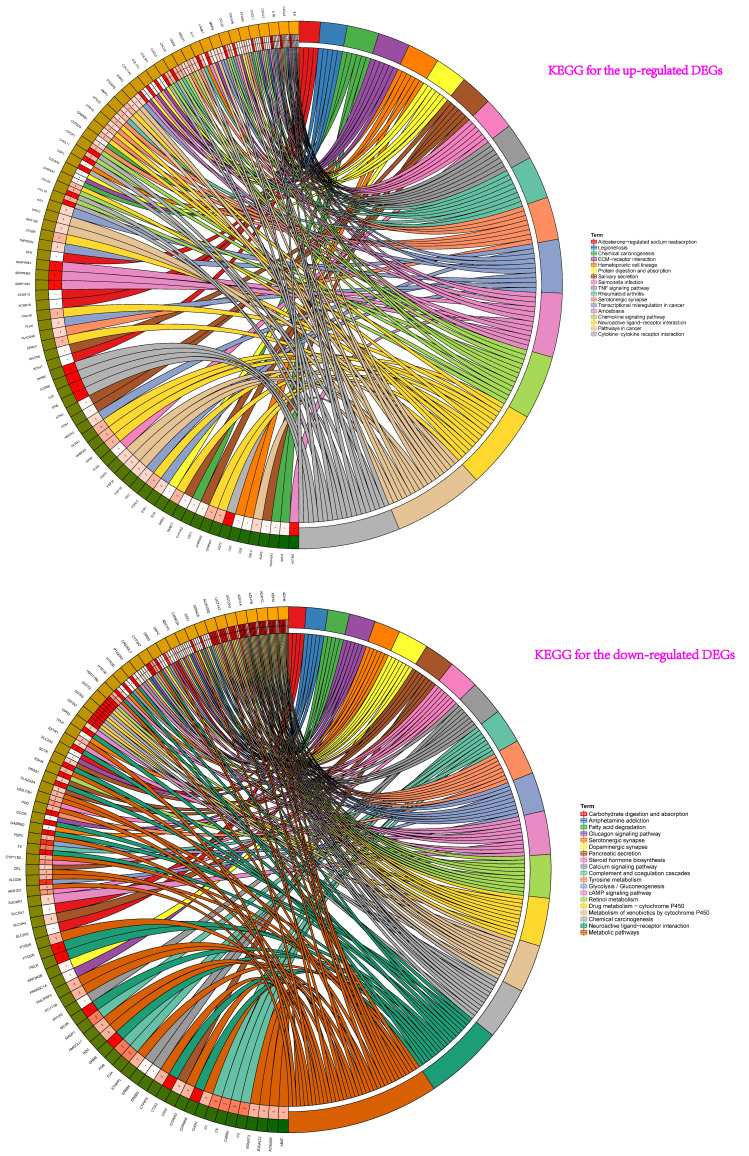
KEGG pathway analyses showed that these up-regulated genes participated in pathway: cytokine-cytokine receptor interaction, amoebiasis, chemokine signaling pathway, neuroactive ligand-receptor interaction, rheumatoid arthritis, pathways in cancer, transcriptional misregulation in cancer, serotonergic synapse, TNF signaling pathway, chemical carcinogenesis et al. For the down-regulated genes of pathway analysis: chemical carcinogenesis, drug metabolism-cytochrome P450, metabolism of xenobiotics by cytochrome P450, retinol metabolism, tyrosine metabolism and neuroactive ligand-receptor interaction et al.

Then, the results of KEGG pathway analysis showed that these up-regulated genes participated in cytokine-cytokine receptor interaction, amoebiasis, chemokine signaling pathway, neuroactive ligand-receptor interaction, rheumatoid arthritis, pathways in cancer, transcriptional misregulation in cancer, serotonergic synapse, TNF signaling pathway, chemical carcinogenesis et al. (Figure 3). For the down-regulated genes, pathway analysis were mainly enriched in chemical carcinogenesis, drug metabolism-cytochrome P450, metabolism of xenobiotics by cytochrome P450, retinol metabolism, tyrosine metabolism, neuroactive ligand-receptor interaction, and glycolysis / gluconeogenesis et al. (Figure 3).

### Integration of the PPI network, module analysis and Potential compounds for treatment

Based on all DEGs, we used the STRING online database to construct PPI networks. The hub-genes were screened by building PPI sub-networks with 12 topological analysis methods. Then we choose the top 15 gene for every topological analysis methods into the Venn plot method, found that 22 hub-genes (C3, CXCL1, CXCL3, CXCL5, CXCL6, CXCL8, CXCL11, CXCR1, CX3CR1, DRD2, FPR2, GNGT1, GNAT3, GRM8, HCAR2, HTR1B, HTR1D, OPRD1, PPY, PTGER3, SSTR1 and SUCNR1) were list in the top, as shown in Figure 4a.

Then, the top five modules (MCODE score > 2) in PPI networks of DEGs were chosen, as shown in Figure 4b. And there are 67 genes in the top five modules including 36 up-regulated and 31 down-regulated genes (supplementary file 1).

To screen out candidate small molecule drugs, CMap database was utilized to analyze consistent differently expressed probesets based on 36 up-regulated and 31 down-regulated genes from the top five modules. CMap predicted the three most significant small molecules (H-9, AZ-628 and phensuximide) as potential therapeutic drugs for ACC. H-9 was the most promising small molecule to reverse the ACC gene expression (Table 2).

### Diagnostic value of hub-genes

**Figure 5:**
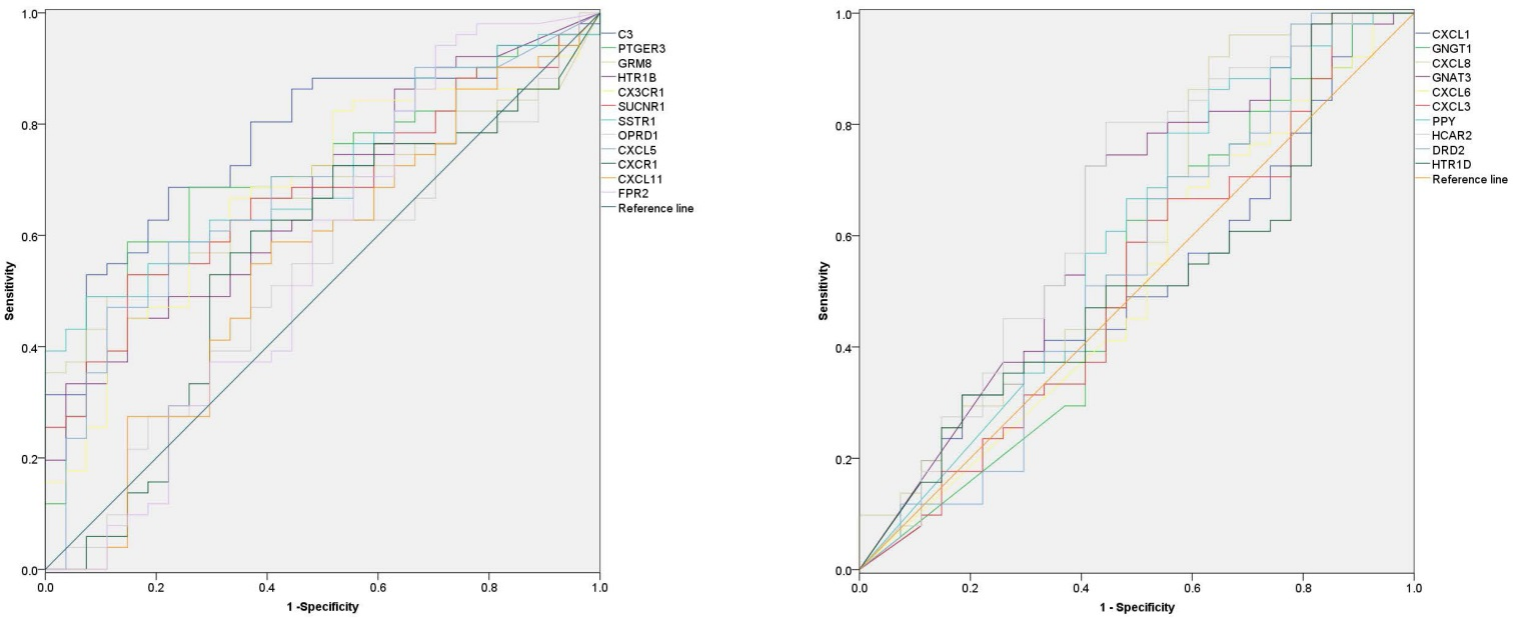
The diagnostic value of 22 hub-genes in distinguishing the death and survival groups, the ROC curve found that nine hub-genes (C3, CXCL5, CX3CR1, GRM8, HCAR2, HTR1B, SUCNR1, PTGER3 and SSTR1) could be used to distinguish the death and survival groups (the p-value for each gene in Table 3).

In order to explore the diagnostic value of 22 hub-genes in distinguishing the death and survival groups, the ROC curve found that nine hub-genes (C3, CXCL5, CX3CR1, GRM8, HCAR2, HTR1B, SUCNR1, PTGER3 and SSTR1) could be used to distinguish the death and survival groups for ACC patients (Figure 5 and Table 3).

### Prognostic significance of hub-genes

As for 22 hub-genes, the prognostic value of them was analyzed by Kaplan-Meier plotter (supplementary file 2). It showed that low mRNA expression of C3 was related with a worse OS, as well as CX3CR1, GRM8, HTR1B, PTGER3, SUCNR1 and SSTR1. Otherwise, high mRNA expressions of four genes (CXCL8, GNAT3, GNGT1 and HCAR2) were related to a worse OS.

As for DFS, low mRNA expressions of three genes (C3, CX3CR1 and SSTR1) were related with worse DFS and high mRNA expressions of four genes (CXCL8, GNGT1, HCAR2 and HTR1D) were related to worse DFS.

### Differential miRNAs expression levels

**Figure 6:**
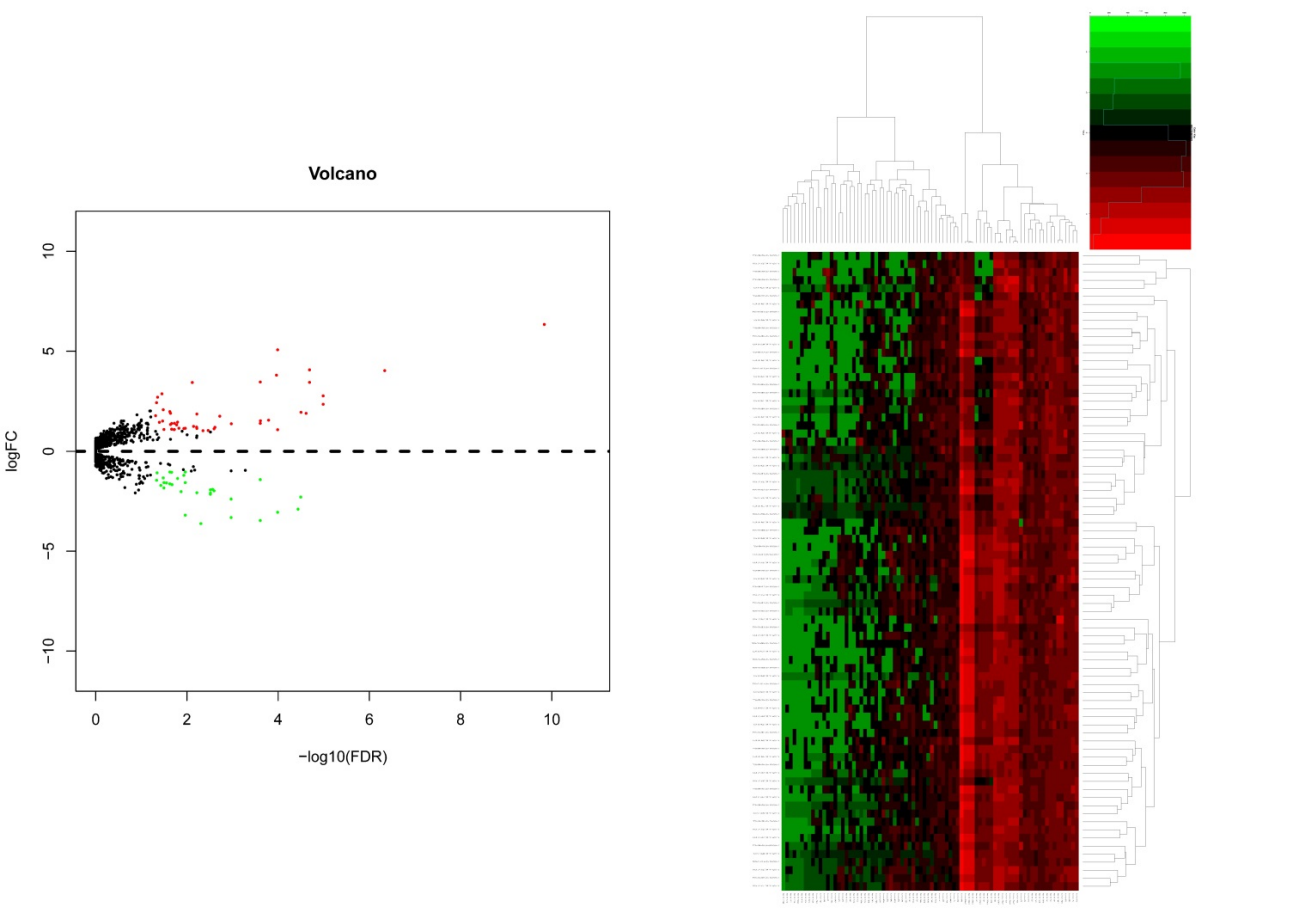
The volcano plot and heatmap of the distribution of differentially miRNAs. Adjust P value <0.05 and |log2FC| >1 was considered the statistical significance of differential miRNAs. 47 genes were up-regulated and 33 genes were down-regulated in dead group compared to alive group.

For miRNAs expression levels, the study included 51 alive samples and 28 dead samples (supplementary file 3). After miRNAs differential expression analysis of microarray data, 47 genes were up-regulated and 33 genes were down-regulated in dead group compared to alive group. The volcano plot and heatmap of the distribution of differentially miRNAs is shown in Figure 6.

### Potential miRNAs involved in regulation of hub-genes

**Figure 7:**
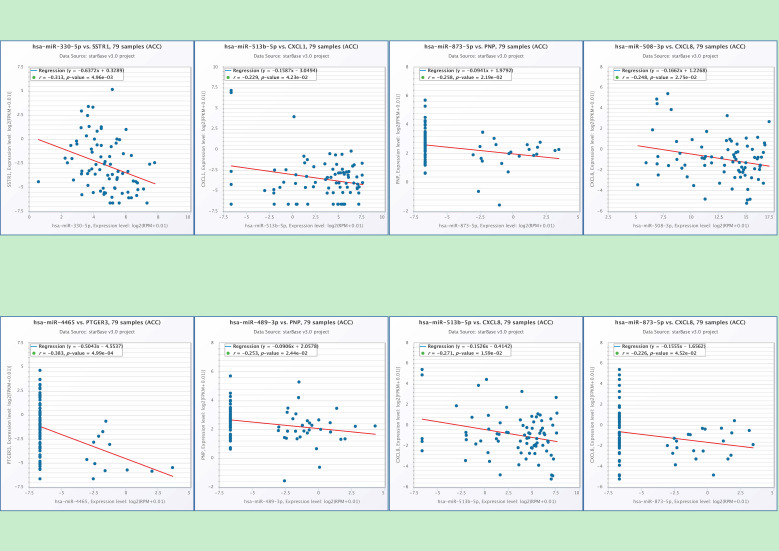
The correlation analysis of hub-genes and predicted miRNAs. There is a clear correlation between hsa-miR-330 and SSTR1 (p=0.005), hsa-miR-513b and CXCL1 (p=0.004), hsa-miR-4465 and PTGER3 (p<0.001), hsa-miR-873 (p=0.02) and PNP, hsa-miR-489 (p=0.02) and PNP, hsa-miR-508 and CXCL8 (p=0.028), hsa-miR-513b and CXCL8 (p=0.016), hsa-miR-873 and CXCL8 (p=0.045).

In the end, we identified six miRNAs that may be involved in regulating the hub-genes through conducting the correlation analysis of hub-genes and predicted miRNAs (Table 4). There is a potential regulatory effect between hsa-miR-330 and SSTR1, hsa-miR-513b and CXCL1, hsa-miR-4465 and PTGER3 (Figure 7). The hsa-miR-873 and hsa-miR-489 may participate in the ACC pathway by regulating PNP (Figure 7). CXCL8 may be regulated by three kinds of miRNAs, such as hsa-miR-508, hsa-miR-513b and hsa-miR-873 (Figure 7). It showed that six miRNAs (hsa-mir-330, hsa-mir-489, hsa-mir-508, hsa-mir-513b, hsa-mir-873, and hsa-mir-4465) may be the play a key role in regulating the hub-genes in the pathways.

### Prognostic significance of six key miRNAs

**Figure 8:**
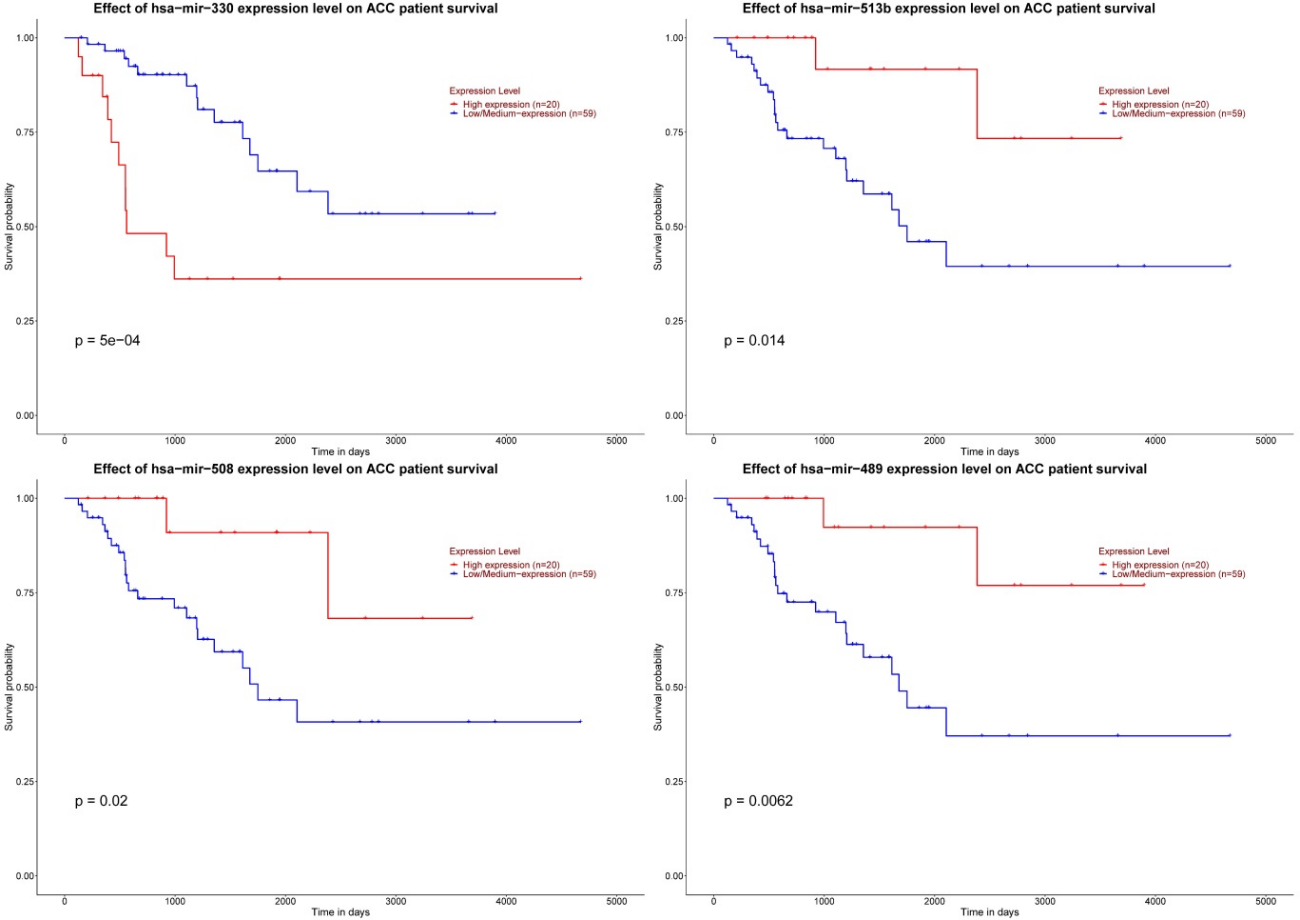
Significant prognosis of four key miRNAs. The P value of four miRNAs is as shown in the figure above, all are < 0.05.

As for six key miRNAs, the prognostic value of these miRNAs was analyzed by Kaplan-Meier plotter. It could be known that low level of hsa-mir-330 was related with worse DFS. Otherwise, elevated levels of three miRNAs (hsa-mir-508, hsa-mir-513b and hsa-mir-489) were related to worse DFS (Figure 8).

## Discussion

The prognosis of ACC is generally very poor, but there are significant individual differences in the progress, recurrence and survival of ACC. Some patients with advanced stages can still achieve longer survival. Advances in genetic analysis technology in the form of next-generation sequencing and the development of bioinformatics tools have helped to study the molecular characterization of these tumors and opened up new research avenues to improve the understanding of patients with this disease, which also could be used to discover different diagnoses and prognosis and therapeutic targets. Compared to alive group, we found the potential biological functions and enrichment pathways in dead group based on 475 up-regulated genes and 354 down-regulated genes. These may be the key pathways leading to the death of different ACC individual patients. Then we used 12 topological analysis methods to choose the most possible 22 hub-gene (C3,CXCL1, CXCL3, CXCL5, CXCL6, CXCL8, CXCL11, CXCR1, CX3CR1,DRD2, FPR2, GNGT1, GNAT3, GRM8, HCAR2, HTR1B, HTR1D, PPY, PTGER3, OPRD1, SSTR1 and SUCNR1) in the role of regulation pathways.

For diagnostic and prognosis significance, it is important for clinical management of ACC patients. The development of genomics methods during the last decade has allowed studying gene expression, genetic and epigenetic alterations at the pan-genomic level in numerous cancer types. Such research results can also be applied to identify tumor subgroups with different biological characteristics and different results in ACC. Based on these as a diagnostic or prognostic molecular marker. For diagnostic value, nine hub-genes (C3, CXCL5, CX3CR1, GRM8, HCAR2, HTR1B, SUCNR1, PTGER3 and SSTR1) could be used to distinguish the death and survival groups for ACC patients. Limited prognostic markers were proposed based on transcriptome studies including SF-1 18, PTTG1 19, EZH2 20 and VAV2 21 et al. We also revealed that mRNA expressions of eleven genes (C3, CXCL8, CX3CR1, GNAT3, GNGT1, GRM8, HCAR2, HTR1B, PTGER3, SSTR1 and SUCNR1) were related to overall survival and expressions of seven genes (C3, CXCL8, CX3CR1, GNGT1, HCAR2, HTR1D and SSTR1) were related to DFS.

Yuan et al. study found that high complement C3 (C3) deposition activates JAK2/STAT3 pathway correlates with in gastric cancer progression and was identified as an independent prognostic factor of poor overall survival 22. C3 plays a role of diagnostic signature including achieved a predictive error of 12.8% and a Generalized Brier Score of 0.108 lung adenocarcinoma and squamous cell carcinoma 23. In addition to the C3, for these genes (CXCL8, CX3CR1, GRM8, HTR1B, HTR1D, PTGER3, SSTR1 and SUCNR1) were related to survival in the ACC and it was also could be useful in other cancers 24-31. GNGT1 and HCAR2 take the lead in be found to have prognostic effects in tumors.

For the treatment of ACC, surgery resection is the only recommended curative methods and complete resection of the primary tumor is predictably associated with a better prognosis and patients in whom there is microscopic or macroscopic involvement of the tumor margins, 5 years survival is as low as 20 and 10%, respectively 32. However, even in patients with complete resection, recurrence and disease progression is still commonly seen, so that adjuvant therapy is frequently recommended. Medical treatment is recommended in patients with stage iii/iv. The mitotane is a compound which combines antitumor and antisecretory effects in order to reduce steroid production by tumor cells. Partial responses were reported in 13% to 33% of cases with response duration of 2 to 190 months. Experience over the years has shown that mitotane does not significantly affect OS, but it can improve DFS 33. Thus, more effective drug development is of particular important. We provide 3 most small molecules (H-9, AZ-628 and phensuximide) as potential therapeutic drugs for ACC. The top compound H-9 is referred to as a potential therapeutic target because it is a PKA inhibitor. However PKA activity has been found to inhibit WNT/beta-catenin-dependent tumorigensis in ACC 34. This is would be an opposite effect, and a possible explanation is that it should be regarded that biological heterogeneity exists in ACC over time. Thus data coming from the primary tumor may not reflect the behaviour of the metastatic tumor 35 that is the most commonly observed tumor setting in the clinical routine.

In recent years, a large number of studies on miRNAs have been conducted worldwide. One miRNA may regulate hundreds of genes at the post-transcriptional level, and one gene can be targeted by multiple miRNAs, leading to the formation of an extremely complex regulatory network 36. It is currently known that it plays an important role in the occurrence and development of a variety of tumors. The uncontrolled expression of miRNAs has been found to be related to the progression and development of various types of human cancers 37. Most miRNAs are directly targeted acts on oncogenes or tumor suppressor genes, thereby participating in various human tumors and their malignant phenotypes. In our current research, we identified 6 miRNAs that may be involved in regulating the hub-genes and high levels of 4 miRNAs (hsa-mir-330, hsa-mir-489, hsa-mir-508 and hsa-mir-513b) were related to the DFS. Similarly, these four miRNAs have also been found to play significant roles in the prognosis, progression and regulation of other tumors 38-42.

However, there were still some shortages and limitations in this study. The samples size is too small, only including 51 alive samples and 27 dead samples with ACC and the conclusions need experimental verification to be firmed and reliable.

## Supplementary Material

Supplementary tables.

## Figures and Tables

**Figure 4a:**
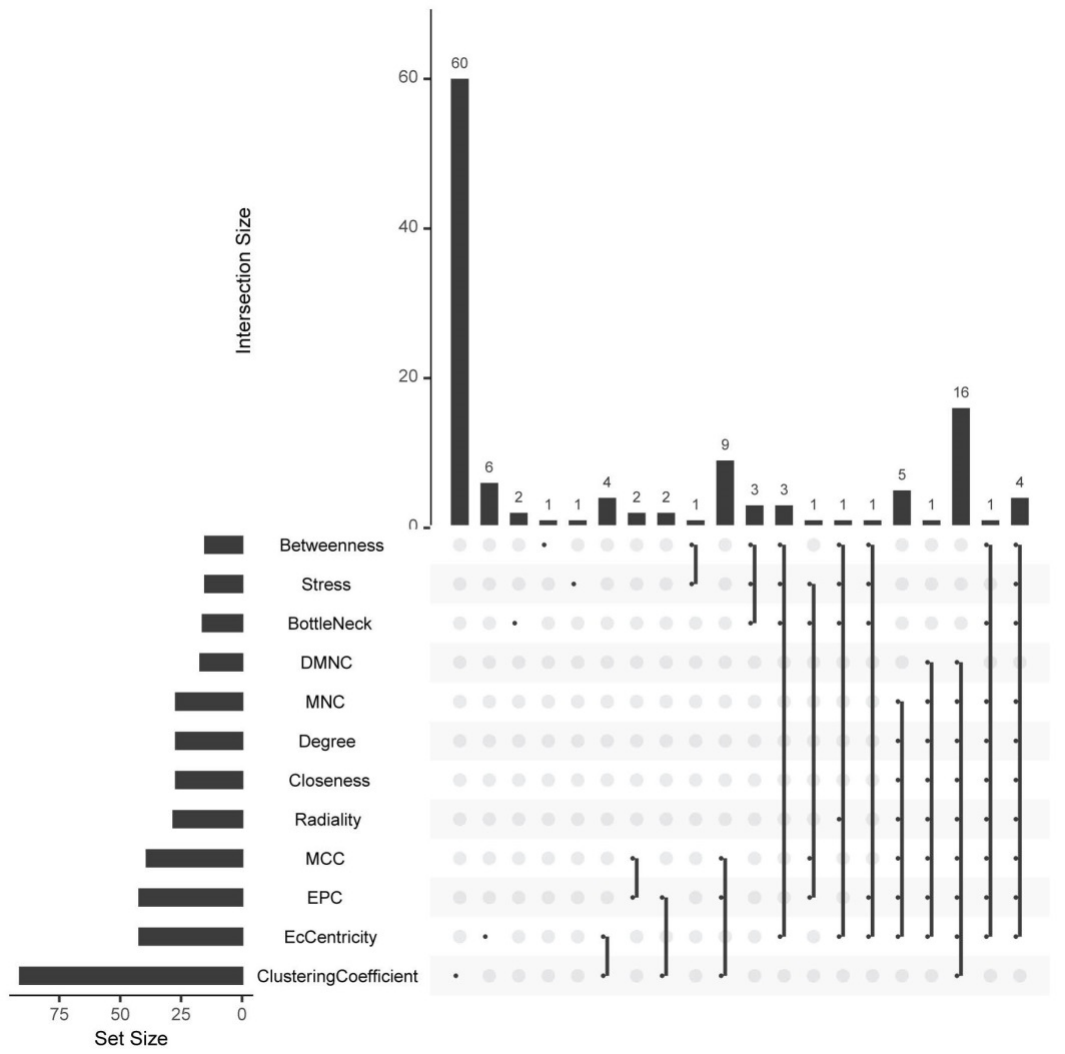
The hub-genes were screened by 12 topological analysis methods. Then choose the top 15 gene for every topological analysis methods into the Venn plot method, found that 22 hub-genes (C3, CXCL1, CXCL3, CXCL5, CXCL6, CXCL8, CXCL11, CXCR1, CX3CR1, DRD2, FPR2, GNGT1, GNAT3, GRM8, HCAR2, HTR1B, HTR1D, OPRD1, PPY, PTGER3, SSTR1 and SUCNR1) were list in the top.

**Figure 4b:**
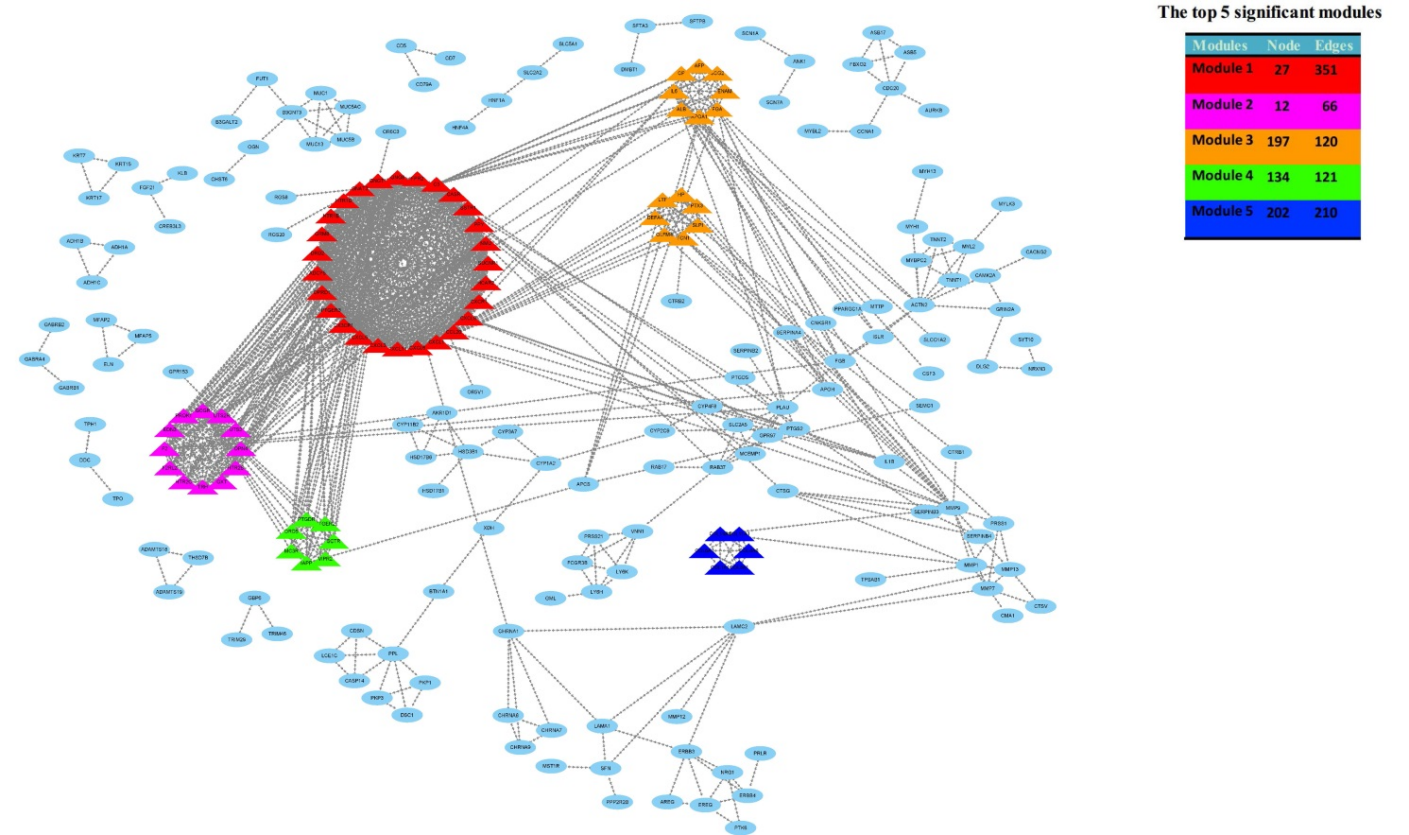
The top five modules (MCODE score > 2) in PPI networks of DEGs were chosen, as shown in Figure 4b. And there are 67 genes in the top five modules including 36 up-regulated and 31 down-regulated genes.

**Table 1:** Characteristics of the patients.

Parameter	Alive (51)	Dead (27)	P
Age (years)	45.96±15.21	49.30±15.89	0.367
Gender			0.851(χ^2^=0.035)
Female	31	17	
Male	20	10	
Stage			0.033(χ^2^=8.745)
T1	7(13.7%)	2(7.4%)	
T2	32(62.8%)	10(37.1%)	
T3	5(9.8%)	4(14.8%)	
T4	7(13.7%)	11(40.7%)	
Lymph node			0.69(χ^2^=8.745)
N0	44(86.3%)	25(92.6%)	
N1	7(13.7%)	2(7.4%)	
Metastasis			0.09(χ2=2.875)
M0	44(86.3%)	19(70.4%)	
M1	7(13.7%)	8(29.6%)	

**Table 2:** The top three compounds identified as treatment options for ACC by CMap analysis.

Rank	Score	ID	Name	Description
1	-96.3	BRD-K70577657	H-9	PKA inhibitor
2	-95.1	BRD-K05804044	AZ-628	RAF inhibitor
3	-90.94	BRD-A18043272	phensuximide	Succinimide antiepileptic

ACC = adrenocortical carcinoma; CMap=connectivity map.

**Table 3:** Receiver Operating Characteristics of the 22 hub-genes.

Genes	AUC	Down(95%CI)	Up(95%CI)	P
**C3**	.772	.667	.877	<0.001
**PTGER3**	.715	.600	.830	.002
**GRM8**	.672	.555	.788	.013
**HTR1B**	.674	.553	.794	.012
**CX3CR1**	.679	.557	.801	.010
**SUCNR1**	.679	.562	.797	.009
**SSTR1**	.712	.600	.824	.002
**OPRD1**	.520	.385	.655	.773
**CXCL5**	.688	.567	.809	.007
**CXCR1**	.562	.423	.701	.369
**CXCL11**	.551	.412	.690	.459
**FPR2**	.558	.411	.706	.401
**CXCL1**	.510	.374	.645	.887
**GNGT1**	.526	.382	.669	.709
**CXCL8**	.605	.465	.745	.129
**GNAT3**	.629	.492	.765	.063
**CXCL6**	.511	.372	.651	.871
**CXCL3**	.516	.375	.656	.821
**PPY**	.590	.449	.731	.195
**HCAR2**	.649	.512	.786	.031
**DRD2**	.547	.403	.691	.498
**HTR1D**	.516	.379	.652	.821

CI, confidence interval; AUC, area under the curve.

**Table 4:** The process of identified six miRNAs that may be involved in regulating the hub-genes (A-D).

Hub-genes (15 up-regulate)	A: the possible miRNAs in ENCORI database (number)	B: miRNA were down-regulated in TCGA (33)		C: Take the intersection of A and B jointly owned	D: correlation analysis of hub-genes and C
CXCL1	83	hsa-mir-653 hsa-mir-1258 hsa-mir-466 hsa-mir-1247 hsa-mir-362 hsa-mir-4501 hsa-mir-489 hsa-mir-194-1 hsa-mir-605 hsa-mir-513a-2 hsa-mir-513a-1 hsa-mir-194-2 hsa-mir-873 hsa-mir-4423 hsa-mir-6842 hsa-mir-876 hsa-mir-585	hsa-mir-99a hsa-mir-6788 hsa-mir-150 hsa-mir-514a-3 hsa-mir-514a-1 hsa-mir-514a-2 hsa-mir-125b-2 hsa-mir-510 hsa-mir-125b-1 hsa-mir-507 hsa-mir-592 hsa-mir-513b hsa-mir-6757 hsa-mir-552 hsa-mir-1287 hsa-mir-508	hsa-mir-873 hsa-mir-489 hsa-mir-362 hsa-mir-513b hsa-mir-653	hsa-mir-513b
CXCL3	101			hsa-mir-873 hsa-mir-489 hsa-mir-362 hsa-mir-150 hsa-mir-653	NO
CXCL5	100			hsa-mir-876 hsa-mir-873 hsa-mir-513b	NO
CXCL6	77			hsa-mir-873 hsa-mir-362	NO
CXCL8	155			hsa-mir-876 hsa-mir-873 hsa-mir-362 hsa-mir-508 hsa-mir-513b hsa-mir-653	hsa-mir-873 hsa-mir-508 hsa-mir-513b
CXCL11	8			0	NO
CXCR1	1			hsa-mir-873	NO
DRD2	0			0	NO
FPR2	16			hsa-mir-362	NO
GNGT1	2			0	NO
GNAT3	0			0	NO
HCAR2	0			0	NO
HTR1D	0			0	NO
OPRD1	0			0	NO
PPY	119			hsa-mir-873 hsa-mir-489	hsa-mir-873 hsa-mir-489
**Hub-genes (7 down-regulate)**	**A: the possible miRNAs in ENCORI database (number)**	**B: miRNA were down-regulated in TCGA (47)**		**C: Take the intersection of A and B jointly owned**	**D: correlation analysis of hub-genes and C**
C3	22	hsa-mir-31 hsa-mir-135b hsa-mir-190b hsa-mir-582 hsa-mir-4465 hsa-mir-372 hsa-mir-214 hsa-mir-223 hsa-mir-130b hsa-mir-548az hsa-mir-7114 hsa-mir-4668 hsa-mir-301b hsa-mir-141 hsa-mir-6720 hsa-mir-3170 hsa-mir-215 hsa-mir-887 hsa-mir-330 hsa-mir-301a hsa-mir-18a hsa-mir-1245a hsa-mir-146b hsa-mir-199b	hsa-mir-222 hsa-mir-205 hsa-mir-4746 hsa-mir-221 hsa-mir-3917 hsa-mir-6783 hsa-mir-6772 hsa-mir-4515 hsa-mir-199a-1 hsa-mir-199a-2 hsa-mir-92b hsa-mir-3662 hsa-mir-675 hsa-mir-937 hsa-mir-499a hsa-mir-1226 hsa-mir-124-2 hsa-mir-7106 hsa-mir-549a hsa-mir-3622a hsa-mir-1269b hsa-mir-371a hsa-mir-124-1	0	NO
CX3CR1	0			0	NO
GRM8	4			0	NO
HTR1B	0			0	NO
PTGER3	132			hsa-mir-371a hsa-mir-214 hsa-mir-675 hsa-mir-4465 hsa-mir-372 hsa-mir-499a hsa-mir-199b	hsa-mir-4465
SUCNR1	0			0	NO
SSTR1	118			hsa-mir-371a hsa-mir-330 hsa-mir-146b hsa-mir-582 hsa-mir-214 hsa-mir-499a	hsa-mir-330

## Abbreviations

C3: complement C3
CXCL1: C-X-C motif chemokine ligand 1
CXCL3: C-X-C motif chemokine ligand 3
CXCL5: C-X-C motif chemokine ligand 5
CXCL6: C-X-C motif chemokine ligand 6
CXCL8: C-X-C motif chemokine ligand 8
CXCL11: C-X-C motif chemokine ligand 11
CXCR1: C-X-C motif chemokine receptor 1
CX3CR1: C-X3-C motif chemokine receptor 1
DRD2: dopamine receptor D2
FPR2: formyl peptide receptor 2
GNGT1: G protein subunit gamma transducing 1
GNAT3: G protein subunit alpha transducin 3
GRM8: glutamate metabotropic receptor 8
HCAR2: hydroxycarboxylic acid receptor 2
HTR1B: 5-hydroxytryptamine receptor 1B
HTR1D: 5-hydroxytryptamine receptor 1D
OPRD1: opioid receptor delta 1
PPY: pancreatic polypeptide
PTGER3: prostaglandin E receptor 3
SSTR1: somatostatin receptor 1
SUCNR1: succinate receptor 1
