# Supplementary material for: The underlying molecular mechanism and drugs for treatment in adrenal cortical carcinoma
Source: Int J Med Sci. 2021 Jun 16;18(13):3026–38. doi: 10.7150/ijms.60261 (PMC8241782; doi:10.7150/ijms.60261)
Supplement: Supplementary file 1 — Supplementary tables. [file ijmsv18p3026s1.pdf]

supplementary file 1

SUID	MCODE_Cluster	MCODE_Node_Status	MCODE_Score	name
81	Cluster 3	Clustered	8	FGA
84	Cluster 2	Clustered	16	F2
92	Cluster 1	Clustered	26	CXCL8
93	Cluster 1	Clustered	26	CXCR1
101	Cluster 5	Clustered	5	COL3A1
102	Cluster 5	Clustered	5	COL1A1
104	Cluster 2	Clustered	16	UTS2R
105	Cluster 2	Clustered	16	UTS2
123	Cluster 3	Clustered	8	ALB
124	Cluster 3	Clustered	8	APOA1
127	Cluster 1	Clustered	26	CXCL6
133	Cluster 1	Clustered	26	CXCL1
134	Cluster 1	Clustered	26	CCL20
144	Cluster 1	Clustered	26	CXCL11
146	Cluster 2	Clustered	16	TRH
147	Cluster 2	Clustered	16	OXT
149	Cluster 3	Clustered	8	IL6
154	Cluster 5	Clustered	5	COL6A3
163	Cluster 1	Clustered	26	CXCL5
165	Cluster 2	Clustered	16	F2RL2
183	Cluster 3	Clustered	8	CP
191	Cluster 1	Clustered	26	CXCL3
202	Cluster 3	Clustered	8	AFP
204	Cluster 3	Clustered	7	HP
223	Cluster 1	Clustered	26	C3
225	Cluster 1	Clustered	26	FPR2
264	Cluster 3	Clustered	7	DEFA4
265	Cluster 3	Clustered	7	LTF
276	Cluster 3	Clustered	7	TCN1
287	Cluster 1	Clustered	26	ADCY5
288	Cluster 1	Clustered	26	DRD2
302	Cluster 4	Clustered	10	DRD5
305	Cluster 3	Clustered	7	OLFM4
307	Cluster 1	Clustered	26	CX3CR1
333	Cluster 1	Clustered	26	GNG8
334	Cluster 1	Clustered	26	GNGT1
339	Cluster 1	Clustered	26	GNAT3
349	Cluster 1	Clustered	26	PTGER3
374	Cluster 3	Clustered	7	SLPI
376	Cluster 2	Clustered	16	HTR2C
378	Cluster 1	Clustered	26	NMU
380	Cluster 1	Clustered	26	GRM8

388	Cluster 1	Clustered	26	PPY
389	Cluster 1	Clustered	26	SSTR1
391	Cluster 5	Clustered	5	COL14A1
396	Cluster 3	Clustered	7	PTX3
403	Cluster 2	Clustered	16	GCGR
404	Cluster 4	Clustered	10	IAPP
426	Cluster 5	Clustered	5	COL11A1
430	Cluster 1	Clustered	26	CASR
437	Cluster 1	Clustered	26	OPRD1
442	Cluster 1	Clustered	26	HTR1D
443	Cluster 1	Clustered	26	HTR1B
468	Cluster 2	Clustered	16	EDN3
469	Cluster 2	Clustered	16	PROK1
493	Cluster 5	Clustered	5	COL4A6
503	Cluster 1	Clustered	26	HCAR2
509	Cluster 4	Clustered	10	VIPR2
544	Cluster 4	Clustered	10	MC3R
546	Cluster 2	Clustered	16	HTR2B
570	Cluster 4	Clustered	10	PTGER2
576	Cluster 4	Clustered	10	SCTR
578	Cluster 1	Seed	26	SUCNR1
595	Cluster 3	Clustered	8	ENAM
612	Cluster 4	Seed	10	PTGDR
647	Cluster 2	Seed	16	OPN4
726	Cluster 3	Seed	8	SCG2

Supplementary file 2. P-value of OS and DFS for the 22 hub-genes.

Genes	OS (P-value)	DFS (P-value)
**C3**	0.02	0.041
**PTGER3**	0.042	0.2
**GRM8**	0.044	0.36
**HTR1B**	0.33	0.41
**CX3CR1**	0.036	0.021
**SUCNR1**	0.046	0.075
**SSTR1**	0.018	0.04
**OPRD1**	0.89	0.86
**CXCL5**	0.13	0.32
**CXCR1**	0.68	0.29
**CXCL11**	0.62	0.66
**FPR2**	0.73	0.34
**CXCL1**	0.7	0.66
**GNGT1**	0.012	0.016
**CXCL8**	0.005	0.032
**GNAT3**	0.047	0.2
**CXCL6**	0.28	0.67
**CXCL3**	0.074	0.32
**PPY**	0.013	0.083
**HCAR2**	0.009	0.012
**DRD2**	0.19	0.3
**HTR1D**	0.38	0.005

OS= overall survival; DFS=disease-free survival.

Supplementary file 3. characteristics of the patients for miRNAs.

Parameter	Alive (51)	Dead (28)	P
Age (years)	45.96±15.21	48.95±15.81	0.37
Gender			0.759(χ^2=0.094)
Female	31	18	
Male	20	11	
Stage			0.03(χ^2=10.749)
T1	7(13.7%)	2(7.1%)	
T2	32(62.8%)	10(35.7%)	
T3	5(9.8%)	5(17.9%)	
T4	7(13.7%)	11(39.3%)	
Lymph node			0.514(χ^2=1.33)
N0	44(86.3%)	26(92.9%)	
N1	7(13.7%)	2(7.1%)	
Metastasis			0.099(χ2=4.617)
M0	44(86.3%)	20(71.4%)	
M1	7(13.7%)	8(28.6%)	
